# The Gift of Sleep

**Published:** 1919-01-25

**Authors:** 


### THE GIFT OF SLEEP.

By A SOCIAL WORKER.

"Night with her train of stars
And her great gift of sleep."

Much attention has been attracted by the lecture given by Miss May Smith during the recent Educational Conferences at University College. The study is a most important item in the larger inquiry into fatigue and its effects upon work, health, and longevity from the physiological and the psychological points of view. Miss Smith has tried upon herself and upon munition workers and others experiments eminently practical. Dr. MacDougal's recording machine tests by the rapid passing before the subject of a paper-tape machine with very small red circles, into each of which an ink dot has to be put. Fatigue evinces itself in a manner easily calculated by dots too few or too frequent. A further test, tried by Miss Smith, was the reading to the subject of forty words connected by varied associations, such as angel, spirit, brandy. In normal conditions about seven mistakes were made, while fatigue, from sleeplessness or other cause, raised the number quickly to thirty. Sometimes both tests were applied together, proving the known psychological fact that attention can be given to two things at once, though a third is excluded. This idea, of course, underlies the use of the rosary, at least in the Roman Church, where meditation is carried out on points apart from the prayers recited and marked by the dropping of each bead. The experiments further showed that while sleeplessness at first made consciousness more intense it presumably reduced rapidly the power of giving attention. A further dose of sleeplessness—only one and a-half hour being spent in sleep on the nights selected—gave new power, but this was quickly exhausted, and the curve fell almost perpendicularly to a point whence restoration to the normal was very slow indeed.

Now this study comes with peculiar interest at a time when it has lately been admitted as a fact that fatigue of itself develops a poisonous toxin. Miss May Smith threw out the suggestion that the long-continued labours of savants and others who take very little sleep may show a power possessed by some human beings of developing an anti-toxin of their own. It may, on the other hand, be found that these people avoid other sources of wastage. They often take little exercise, they attend to no family business—if, indeed, they have a family, they are apt to shirk its responsibilities. They seldom, in fact, fatigue themselves over anything but the one subject of concentration. But in education and home management some clear knowledge on this subject would be very helpful. Uneducated people have little idea that children need more sleep than grown-up people, and will let quite tiny ones play in the street till 11.30 on summer nights, and even stay up in the kitchen in winter till disposed to go to bed themselves. Sometimes Nature asserts itself. The present writer knows one neglected family where one or other child will run in hot and grimy from the street, throw itself down on the coverless mattress of the family bed, and obey the impulse to sleep. Many slum children again spend their short nights in a room that has not been aired, a bed that has not been made, and the clothes they have worn all day. If, in addition, as is only too likely, they are verminous, such children may well be found asleep in school next day; very probably they are pitied there as breakfastless, having been utterly disinclined for any food before going out into the air. These are familiar cases. What we want to ask is this: "Does fatigue toxin develop in these children and lead to wasting, to feebleness of mind, or to a condition favourable to tuberculosis?" And "is it not possible that what used to be called 'growing pains' and are now called rheumatic ones are really pains caused by the working of this toxin in the system?" They are noticeably common among children of excitable or "clever" tendencies and frail physique.

The tendency in schools, other than the elementary, constantly to lengthen holidays and shorten terms, while adding, besides an ever-widening curriculum, such activities as debating societies, a school magazine, school theatricals, charitable undertakings, leaves a mass of children terribly exhausted at the end of terms, and causes many others to sleep badly during term-times. If school work were less compressed, the holidays would still be amply long. They are so long now that the power of resistance to work has to be laboriously relearnt at the beginning of each term. And violent study is apt to be relieved by equally violent play. Some move seems likely to be made against the hour or two of before-breakfast work which is so unspeakably loathed by the inmates of boarding schools, and is probably of little use to their work. A re-adjustment of hours and meals might allow of at least a twenty minutes' nap or rest after a dinner at perhaps twelve o'clock. There are, of course, some children who show an overweening sleepiness, perhaps, like the Fat Boy of *Pickwick* from over-eating, perhaps from a tendency to brain anæmia, or again from sluggish liver, or from merely bad habits of idleness. Such cases want individual attention.

Meantime health visitors may well do more in friendly chatting with mothers about the children's sleep. One mother lately remarked that putting a child down for a nap is a good way of getting your hands free when you are busy. And among causes for improvement in children received at the St. Pancras School for Mothers Day Nursery "more sleep" is expressly mentioned.

Miss May Smith made an interesting note of the fact that she found irritability increase under the influence of want of sleep, and take the form at times of "being rather easily offended." Here is a valuable psychological note. The capacity to give full attention to work is the special point under study, it is found to diminish according to a ratio of fatigue; may it not be the case that "queer-tempered" children need less brain work, shorter walks, or less vigorous games than others, or, of course, more sleep? The amount of sleep required by an adult for good health and peace of mind seems to vary with the individual. We shall be grateful to those who will make further experiments in the matter for our guidance, and hope they will further study degrees of sleep. Some sub-epileptic subjects suffer from slumber so deep as to be dangerous, and sometimes a cause of most unpleasant habits, and we all know the practical difference between refreshing and unrefreshing sleep. Altogether the subject is both attractive and important, and a fruitful field for the research worker. It would have been very interesting to test for fatigue toxin some of the overworked school children whose cases were lately exposed. The boy of eleven, for instance, in a house not fifty miles from London, who while attending school worked forty-nine and a-half hours weekly as barber's lather boy would have been an interesting study.

